# Applicability of Time-Averaged Holography for Micro-Electro-Mechanical System Performing Non-Linear Oscillations

**DOI:** 10.3390/s140101805

**Published:** 2014-01-21

**Authors:** Paulius Palevicius, Minvydas Ragulskis, Arvydas Palevicius, Vytautas Ostasevicius

**Affiliations:** 1 Department of Mathematical Research in Systems, Kaunas University of Technology, Studentu st. 50-222, Kaunas LT-51368, Lithuania; E-Mail: minvydas.ragulskis@ktu.lt; 2 Faculty of Mechanical Engineering and Mechatronics, Kaunas University of Technology, Kestucio St. 27, Kaunas LT-44312, Lithuania; E-Mail: arvydas.palevicius@ktu.lt; 3 Institute for Hi-Tech Development, Faculty of Mechanical Engineering and Mechatronics, Kaunas University of Technology, Student St. 65, Kaunas LT-51368, Lithuania; E-Mail: vytautas.ostasevicius@ktu.lt

**Keywords:** cantilever, chaos, holography

## Abstract

Optical investigation of movable microsystem components using time-averaged holography is investigated in this paper. It is shown that even a harmonic excitation of a non-linear microsystem may result in an unpredictable chaotic motion. Analytical results between parameters of the chaotic oscillations and the formation of time-averaged fringes provide a deeper insight into computational and experimental interpretation of time-averaged MEMS holograms.

## 1. Introduction

Interferometry is a powerful experimental technique for the analysis of profiles of surfaces, detection of deflections, motion and structural vibrations of microsystems, where the amplitudes of those vibrations are in the range of nanometers to a few micrometers [1–4]. Various applications of this experimental method are being developed. Analysis of viscous and material damping in microstructures by means of interferometric microscopy [5], interferometric readout of multiple cantilever sensors in liquid samples [6], interferometric study of reliability of microcantilevers [7] are typical examples of applications of interferometry in microsystems analysis.

Holographic interferometry is a powerful optical method for mapping changes in the shape of three-dimensional objects with high accuracy. Digital holography is used for nondestructive testing, strain analysis and analysis of vibrations [8] in microsystems. An overview of a large variety of its applications for MEMS characterization, residual stress measurement, design and evaluation, and device testing and inspection is given in [9]. Digital holography and speckle interferometry are also widely used for the quality inspection and the assessment of reliability of microsystems. These optical techniques are employed for the measurement of displacements, deformations induced by mechanical, thermal, or electrostatic loads [10,11].

Time average holography is an experimental method for the quantitative registration of surface oscillations, which has been widely applied to the investigation of microsystems. The application of this method has been employed in dynamic micro-metrology [12], dynamic characterization of MEMS diaphragm [13], measurement of static and vibrating microsystems [14].

One of the assumptions made when applying the time-average holography method is that oscillations are harmonic, which might not be the case in real life applications of microsystems. It is well known that even a periodic excitation of non-linear system may result in unpredictable chaotic behavior. Nonlinear and chaotic effects in microsystems are widely investigated in [15–18]. Nonlinear dynamic and chaotic behavior of electrostatically actuated MEMS resonators subjected to random disturbance are investigated analytically and numerically in [15].

Computation and plotting of patterns of time average holographic fringes in virtual numerical environments involves such tasks as modeling of the optical measurement setup, geometrical and physical characteristics of the investigated structures and the dynamic response of analyzed microsystems [19]. Holographic interferometry, being a non-destructive whole field technique capable of registering oscillations of micro-components, cannot be exploited in a straightforward manner [20]. There exist numerous numerical techniques for interpretation of patterns of fringes in the registered holograms of different oscillating objects and surfaces. Unfortunately, sometimes straightforward application of these motion reconstruction methods (for example ordinary fringe counting technique, *etc.)* does not produce acceptable and interpretable results.

A fixed-fixed paradigmatic fixed-fixed beam model is used to illustrate the formation of time-averaged holographic fringes when the beam performs complex transient oscillations. A typical MEMS device comprising a deformable fixed-fixed beam over a fixed ground electrode is modeled. Finite element method (FEM) is used for the simulation of this MEMS device using a COMSOL Multiphysics package.

This article is organized as follows. Optical background of the formation of time-averaged holographic interference fringes is given in Section 2. A description of the model for the fixed-fixed beam is given in Section 3. Numerical results for the fixed-fixed beam with different conditions are given in Section 4. The description of the process of computational reconstruction of time-averaged holographic fringes and discussion on various problems when beam performs chaotic oscillations is given in Section 5. The details of the experiment are given in Section 6. Conclusions are given in Section 7.

## 2. Optical Background

**Figure 1.**
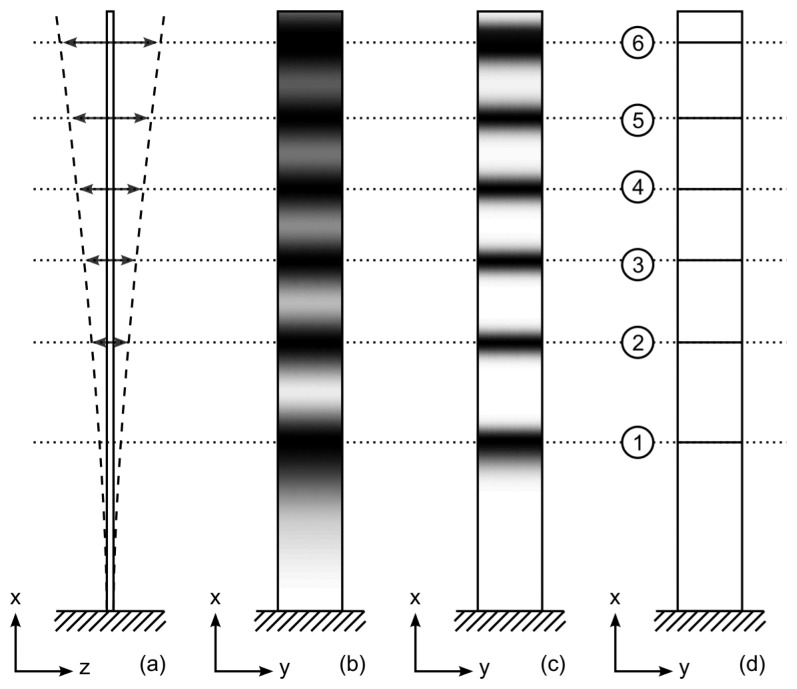
A schematic diagram illustrating the formation of time-averaged holographic fringes: the one-dimensional structure oscillating according to its first eigenform is shown in part (**a**); corresponding gray-scale image in the holographic plane is represented in part (**b**); the contrast enhanced time-averaged hologram is shown in part (**c**); centerlines of time-averaged fringes and their consecutive numbers are represented in part (**d**).

The basic principle of the formation of time-averaged holographic interference fringes can be illustrated by the harmonically oscillating cantilever beam example (Figure 1). Let us assume that the harmonic vibration of the beam is defined as

**Figure 2.**
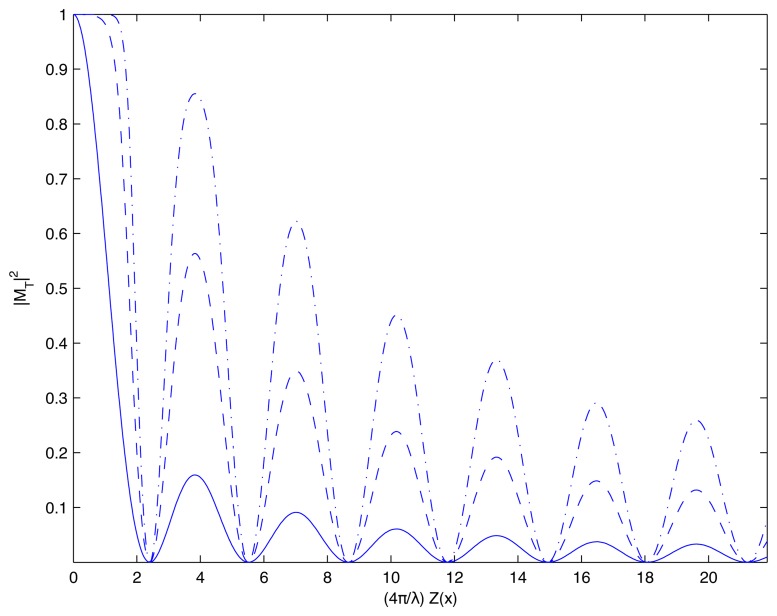
Contrast enhancement of time-averaged holographic fringes: the solid line represents the decay of intensity at increasing amplitude *Z* (*x*); the dashed line shows mapped intensity levels at *k* = 4 (dashed line) and *k* = 8 (dash-dotted line).

(1) Z(x)sinωt

where *t* is time; *x* is the longitudinal coordinate of the beam; *Z* (*x*) is transverse amplitude of oscillations of the one-dimensional beam at coordinate *x; ω* is the frequency of harmonic oscillations. Then the characteristic function defining the complex amplitude of the laser beam *M_T_* in the plane of the hologram takes the form [21]:

(2) $$M_{T}=\underset{T\to \infty }{\lim}\frac{1}{T}\int_{0}^{T}\exp\left(i\left(\frac{4\pi }{\lambda }\right)Z(x)\sin\omega t\right)dt=J_{0}\left(\left(\frac{4\pi }{\lambda }\right)Z(x)\right)$$

where *T* is the exposure time of the hologram, (T ≫ 1/*ω*); λ is the laser wavelength; and *J*_0_ is the zero order Bessel function of the first kind. Then, the resulting intensity *I* of the point (*x*, *y*) on the hologram is

(3) $$I(x,y)=a^{2}(x,y){\left|M_{T}\right|}^{2}$$

where *a*(*x*, *y*) is the distribution of the amplitude of the incident laser beam. A schematic diagram of a cantilever beam oscillating according to the first eigenform is presented in Figure 1a; the time-averaged pattern of holographic interference fringes is illustrated in Figure 1b. Note that the decay of gray-scale intensity is rather fast at increasing amplitudes of oscillation. Better visualization of higher order time-averaged fringes requires contrast enhancement of the time-averaged image. As a limited number of intensity levels is used for the digital representation of images a sigmoid mapping function can be used to distort the intensity scale for better visualization of the results of calculations:

(4) $$F(I)=\frac{\tanh(kI)}{\tanh(k)}$$

where parameter *k* characterizes the level of distortion, 0 < *k* < ∞. Figure 2 illustrates the decay of gray-scale intensity without (the solid line) and with intensity mapping at *k* = 2 (the dashed line). The contrast enhanced time-averaged pattern of holographic fringes at *k* = 4 (*k* = 8) is illustrated in Figure 1c. The identification of fringes centerlines and employment of fringe counting techniques results into Figure 1d. Note, that schematic illustration presented in Figure 1 is based on one-dimensional structure, therefore finite width along the y-axis is used only for illustrative purposes.

The ability to enumerate time-averaged holographic fringes and to identify their centerlines results into accurate reconstruction of the field of amplitudes of harmonic oscillations. Really, the amplitude of harmonic oscillations at point *x_k_* corresponding to centerline of the k-th time-averaged holographic fringe equals to:

(5) $$Z(x_{k})=\frac{\lambda r_{k}}{4\pi }$$

where *r_k_*; *k* = 1, 2, … is the *k*-th root of the zero order Bessel function of the first kind. Note that the uncertainty of such a reconstruction is directly related to density of time-averaged fringes in the observation window. On the other hand, the density of time-averaged fringes is directly related to amplitudes of harmonic osculation and laser wavelength. It would be difficult to reconstruct the field of amplitudes from one time-averaged fringe. These constrains define limits of applicability of time-averaged laser holography in engineering applications. The laser wavelength if usually a fixed constant (λ = 632.8 *nm*, for HeNe laser). Thus one must pre-assess the results of the optical experiment.

All schematic diagrams in figure are based on the assumption that the illumination and the observation directions are perpendicular to the plane of the beam in the state of equilibrium (in the par-axial model). In general case the distribution of intensity of the laser beam *a*^2^ (*x*, *y*) reads [21]:

(6) $$a^{2}(x,y)=I_{L}\left(k_{d}(NL)+k_{s}{\left(VR\right)}^{n}\right)$$

where *I_L_* is the intensity of the incident laser beam, *k_d_* is the diffuse reflection coefficient, *k_s_* is the specular reflection coefficient, *n* is a coefficient describing the smoothness of the surface, *V* is the direction of the viewer, *R* is the direction of the reflection. The pattern of time-averaged holographic fringes does not depend on the static deformations of the structure, nor from the distance between the structure and the hologram.

## 3. Fixed-Fixed Cantilever Beam Model

**Figure 3.**
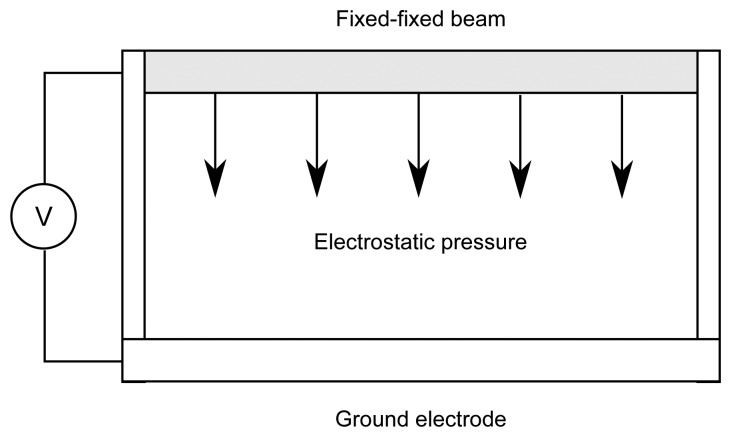
The schematic diagram of the MEMS device—a fixed-fixed beam with an applied voltage *V* over a ground plane.

As mentioned in the introduction we will use fixed-fixed beam model for the computational experiment illustrating the formation of time-averaged holographic fringes when the beam performs complex transient oscillations. A schematic diagram of a typical MEMS device comprising a deformable fixed-fixed beam over a fixed ground electrode is shown in Figure 3. Potential difference is applied between the beam and the ground electrode which induces electrostatic charges. These two electrostatic charges creates electrostatic pressure normal to the surface of conductor.

**Figure 4.**
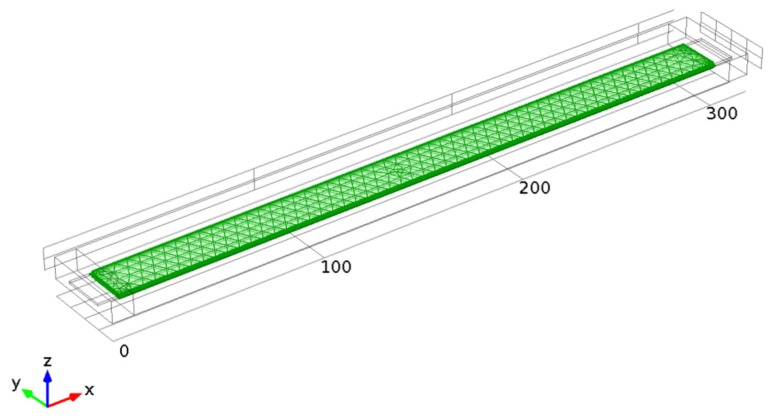
Fixed-fixed beam MEMS model geometry and its FEM mesh.

Finite element method (FEM) is used for the simulation of this MEMS device. Time-dependent simulation is implemented in COMSOL Multiphysics package; Figure 4 provides the geometry and meshing of the MEMS device.

A material used for the beam structure is Silicon with relative permittivity *ε* = 11.7, density *ρ* = 2,329 kg/m^3^, Young's modulus *E* = 170 e9 Pa and Poisson's ratio *v* = 0.28. The polysilicon is assumed to be heavily doped, so that electric field penetration into the structure can be neglected. The beam resides in an air-filled chamber that is electrically insulated. The dimensions of the beam are: Length 300 μm; Depth 20 μm; and Height 1 μm. The model considers a layer of air 6 μm thick above and 10 μm thick to the sides of the beam, and the air gap between the bottom of the beam and the grounded plane is initially set to 3 μm. An unstructured tetrahedral mesh is used for the fixed-fixed beam and its surroundings.

Electromechanics physics Comsol interface is used for computational simulation. It combines solid mechanics and electrostatics with a moving mesh to model the deformation of electrostatically actuated structures:

(7) $$\rho \frac{\partial^{2}u}{\partial t^{2}}-\nabla \cdot \sigma =F\upsilon$$

(8) $$\nabla \cdot D=\rho_{\upsilon }$$

here *u* denotes the displacement field; *t* is time; *ρ* is the material density; *σ* is the stress tensor; *F* is an external volume force; *D* is electric displacement or electric flux density; *υ* is velocity and *ρ_υ_* is charge density in vacuum.

An electrostatic force caused by the potential difference between the two electrodes bends the beam toward the grounded plane beneath it. This model calculates the electric field in the surrounding air in order to compute the electrostatic force. As the beam bends, the geometry of the air gap changes continuously, resulting in a change in the electric field between the electrodes. The coupled physics is handled automatically by the Comsol electromechanics interface.

The electrostatic field in the air and in the beam is governed by Poisson's equation:

(9) −∇⋅(ɛ∇V)=0

where derivatives are taken with respect to the spatial coordinates. The force density that acts on the electrode of the beam results from Maxwell's stress tensor:

(10) $$F_{es}=-\frac{1}{2}(E\cdot D)n+(n\cdot E)D$$

where *E* and *D* are the electric field and electric displacement vectors and *n* is the outward normal vector of the boundary. Note that this force is always oriented along the normal of the boundary.

A condition of the zero charge on the boundary is used as this is the default boundary condition at exterior boundaries.

(11) −n⋅D=0

The initial values for electric potential and solid displacement field are:

(12) $$u{|}_{t=0}=0,V{|}_{t=0}=0$$

The fixed constraint condition that makes the geometric entity fixed (the displacements are zero in all directions) are applied to the edges of the beam. Ground boundary condition is applied to the bottom plain which gives zero potential on the boundary. The boundary condition is applied to fixed-fixed beam of an electric potential *V*_0_.

(13) $$V=V_{0}$$

## 4. Computational Results for a Fixed-Fixed Cantilever Beam

**Figure 5.**
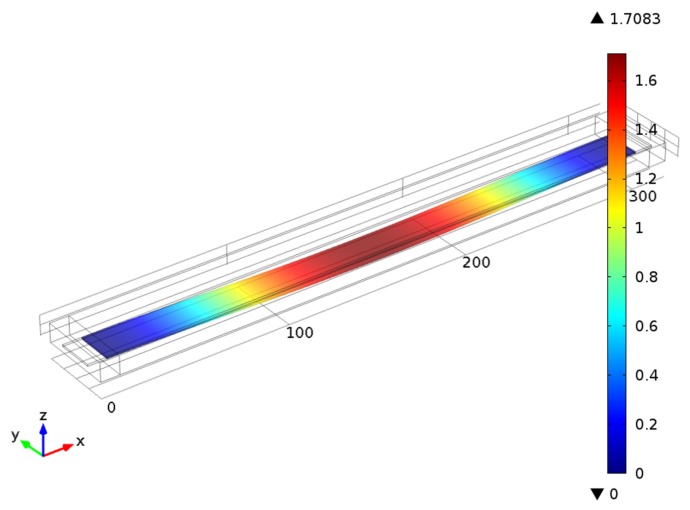
The shape of the fixed-fixed beam in the state of maximum deformation while performing periodic oscillations according to the first computational experiment.

Computational simulation is performed for the model of fixed-fixed beam (the model is described in details in Section 3) with three different electric potential boundary conditions. In order to simplify the visualization of transient processes we select a single point at the center of the beam (where the displacements are largest) and plot time signals and phase diagrams for all three different potential boundary conditions. The illustration of the deformed fixed-fixed beam is given in Figure 5.

During the first computational experiment (i) a DC (direct current) bias of *V_DC_* = 40 *V* is applied to the micro-device in order to obtain the system's response. The DC bias is applied using the standard Comsol smoothed step function which allows to increase the voltage incrementally from 0 to *V_DC_* during the transient interval ranging from 0 to 6e−6. After the transient interval the DC bias is set to a constant:

(14) $$V=V_{DC}$$

The second computational experiment (ii) employs a DC bias on top of a sinusoidal AC voltage:

(15) $$V=V_{DC}+V_{AC}\sin(2\pi ft)$$

where *V_DC_* = 38 *V* and *V_AC_* = 2 *V*. Finally, a DC bias of *V_DC_* = 35 *V* with AC voltage of *V_AC_* = 5 *V* is applied in the last computational experiment (iii). The exposure time *t* used in all simulations is ranging form 0 to 1*e* − 4 with a time step of 2*e* − 7*s*.

**Figure 6.**
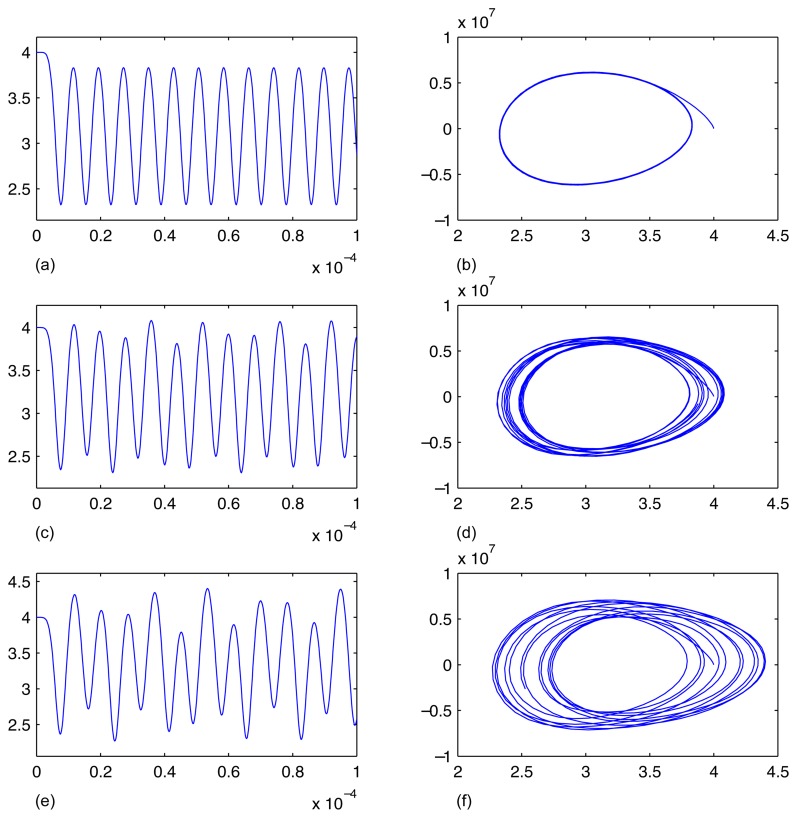
Results of simulations illustrating the dynamics of central node of the fixed-fixed beam. Parts (**a**) and (**b**) show the transient process in time and in phase diagram when the micro-device is excited according to the first computational experiment. Parts (**c**) and (**d**) ((**e**) and (**f**)) illustrate the results of the second (the third) computational experiments.

The results of computational experiments are presented in Figure 6. It can be seen that the response of the system is periodic under the constant DC bias excitation. Note that these periodic oscillations are caused not by a harmonic excitation - the beam is deflected from the state of equilibrium during the short transient process and continues to oscillate because the damping is small (mass damping parameter *α_dM_* = 418.9 [1/*s*] and stiffness damping parameter *β* = 8.29*e* − 13*s*).

The results of the second computational experiment are different compared to the periodic oscillation caused by the short DC transient in the first computational experiment. The harmonic AC component interacts with the natural frequency of the beam - the resultant process of oscillations is no longer periodic (Figure 6c,d). Finally, the response of the system to last excitation is clearly nonlinear. The attractor in the phase plane diagram looks like a chaotic attractor even in a relatively short observation window (Figure 6f).

## 5. Computational Reconstruction of Time-Averaged Holographic Fringes

Time-averaged holographic fringes illustrated in Figure 3 are based on the assumption that structure performs harmonic oscillations. Dynamics analysis of the fixed-fixed beam MEMS shows that transient processes can be very complex. Even in the most simple case periodic oscillations of the beam are not harmonic. This fact requires a separate analysis of processes governing the formation of time-averaged holographic fringes in the hologram plane.

### 5.1. Computational Reconstruction of Time-Averaged Holographic Fringes

The characteristic function defining the complex amplitude of the laser beam *M_t_* when the according surface point performs oscillations defined by function *ξ* (*t*) reads:

(16) $$\begin{array}{c} M_{T}=\underset{T\to \infty }{\lim}\frac{1}{T}\int_{0}^{T}\exp\left(\iota \frac{4\pi }{\lambda }\xi (t)\right)dt=\\ \underset{T\to \infty }{\lim}\frac{1}{T}\int_{0}^{T}\cos\left(\frac{4\pi }{\lambda }\xi (t)\right)dt+\underset{T\to \infty }{\iota \lim}\frac{1}{T}\int_{0}^{T}\sin\left(\frac{4\pi }{\lambda }\xi (t)\right)dt=\\ R_{c}+\iota R_{s} \end{array}$$

Note that when *ξ* (*t*) = *Z* sin (*ωt*) the complex part *R_s_* tends to zero because integrant is an odd function. Then *M_t_* can be computed as an arithmetic average over discrete time steps in a period of harmonic oscillations:

(17) $$\begin{array}{c} M_{T}=\underset{T\to \infty }{\lim}\frac{1}{T}\int_{0}^{T}\cos\left(\frac{4\pi }{\lambda }Z\sin(\omega t)\right)dt=\\ \underset{T\to \infty }{\lim}\overset{n}{\underset{k=1}{\text{∑}}}\cos\left(\frac{4\pi }{\lambda }Z\sin\left(\left(k-1\right)\frac{2\pi }{k}\right)\right)dt=J_{0}\left(\frac{4\pi }{\lambda }Z\right) \end{array}$$

But when oscillations are defined by a non-harmonic function *ξ* (*t*) *R_s_* does not converge to zero and the gray-scale level in the hologram plane reads (assuming that *a*^2^ (*x*, *y*) = 1):

(18) $${\left|M_{T}\right|}^{2}=R_{c}^{2}+R_{s}^{2}$$

In practice one needs to compute arithmetic averages of *R_c_* and *R_s_* over a sufficiently long set of discrete points in the exposure time interval.

### 5.2. Time-Averaged Holographic Fringes Induced by Chaotic Oscillations

It is well known that a non-linear system with a harmonic load may exhibit complex chaotic solutions.

Lets denote intensity level of interference bands as stochastic time series defined by process *ξ* (*t*). If given time series *ξ_i_* is normally distributed with variance *σ*^2^ then the intensity level of interference bands decrease exponentially.

(19) $$\begin{array}{c} E\xi^{2k-1}\equiv 0,k=1,2,\ldots \\ E\xi^{2k}\equiv 1\cdot 2\cdot \ldots \cdot (2k-1)\sigma^{2k}=(2k-1)!!\sigma^{2k},k=1,2,\ldots \\ {\left|M_{T}\right|}^{2}=\underset{m\to \infty }{\lim}\left({\left(\frac{1}{m}\overset{m}{\underset{i=1}{\text{∑}}}\cos\left(\frac{2\pi }{\lambda }\xi_{i}\right)\right)}^{2}+{\left(\frac{1}{m}\overset{m}{\underset{i=1}{\text{∑}}}\sin\left(\frac{2\pi }{\lambda }\xi_{i}\right)\right)}^{2}\right)=\\ \underset{m\to \infty }{\lim}{\left(\frac{1}{m}\overset{m}{\underset{i=1}{\text{∑}}}\overset{+\infty }{\underset{k=0}{\text{∑}}}\frac{{\left(-1\right)}^{k}{\left(\frac{2\pi }{\lambda }\xi_{i}\right)}^{2k}}{\left(2k\right)!}\right)}^{2}=\\ {\left(\overset{+\infty }{\underset{k=0}{\text{∑}}}\frac{{\left(-1\right)}^{k}{\left(\frac{2\pi }{\lambda }\right)}^{2k}}{\left(2k\right)!}\underset{m\to \infty }{\lim}\overset{m}{\underset{i=1}{\text{∑}}}\frac{{\left(\xi_{i}\right)}^{2k}}{m}\right)}^{2}=\\ {\left(\overset{+\infty }{\underset{k=0}{\text{∑}}}\frac{{\left(-1\right)}^{k}{\left(\frac{2\pi }{\lambda }\right)}^{2k}}{\left(2k\right)!}\cdot \left(2K-1\right)!!\cdot \sigma^{2K}\right)}^{2}={\left(\overset{+\infty }{\underset{k=0}{\text{∑}}}\frac{{\left(-1\right)}^{k}{\left(\frac{2\pi }{\lambda }\sigma \right)}^{2k}}{\left(2k\right)!!}\right)}^{2}=\\ {\left(\overset{+\infty }{\underset{k=0}{\text{∑}}}\frac{{\left(-1\right)}^{k}{\left(\frac{2\pi }{\lambda }\sigma \right)}^{2k}}{\left(2^{k}k\right)!}\right)}^{2}={\left(\overset{+\infty }{\underset{k=0}{\text{∑}}}\frac{{\left(-1\right)}^{k}}{k!}{\left(\frac{1}{2}{\left(\frac{2\pi }{\lambda }\sigma \right)}^{2}\right)}^{k}\right)}^{2}=\\ \exp^{2}\left(-\frac{1}{2}{\left(\frac{2\pi }{\lambda }\sigma \right)}^{2}\right) \end{array}$$

The importance of this derivation cannot be overestimated—time-averaged holographic fringes do not form when the oscillation of the investigated object is chaotic. One should make sure that chaotic oscillations do not affect the components of the experimental setup before questioning the operability of the optical system—if only time-averaged hologram does not produce any fringes.

### 5.3. The Formation of Interference Fringes

So far the dynamics of the central point of the fixed-fixed beam is discussed (where the deflections from the state of equilibrium are largest). In order to visualize the dynamic of the whole surface of the beam we will use whole field Time-Averaged holography simulation techniques.

FEM modeling yields complete data on the dynamics of the fixed-fixed beam-distances between the beam plane and the ground plane are available from the simulation results for all time steps. The method described in the Sections 2 and 5.1 will be applied for the data produced by all three computational experiments. Equation (3) is used for each point on the plane in order to determine the gray-scale level in time-averaged hologram. Laser wavelength used for calculation is set to λ = 632.8 nm. Other parameters are set to *I_L_* = 1; *k_d_* = 1; *k_s_* = 0. Note that we model a non-specular surface of the beam.

**Figure 7.**
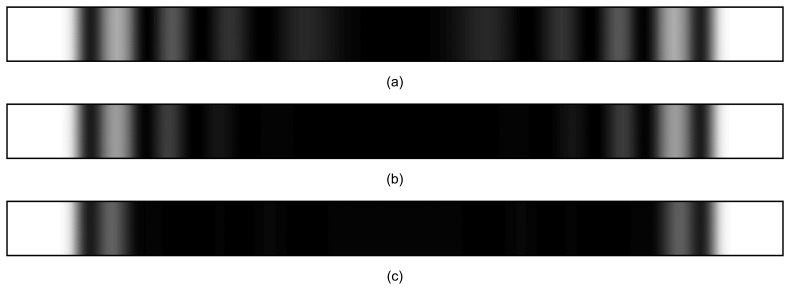
The formation of interference fringes for: (**a**) computational experiment (i); (**b**) computational experiment (ii); (**c**) computational experiment (iii).

Numerically reconstructed time-averaged holograms generated by the fixed-fixed beam according to the computational experiments (i), (ii) and (iii) are illustrated in Figure 7a,b and c. Note that time-averaged fringes in part b are less developed compared to part a. This can be explained by a much more complex transient process the beam is experiencing under excitation (ii). It is almost impossible to observe time averaged fringes in part c—complex chaotic oscillations do not generate time-averaged holographic fringes (these results correspond well to the analytical conclusions in Section 5.2.

### 5.4. The Formation of Interference Fringes for Periodic and Harmonic Oscillation Laws

**Figure 8.**
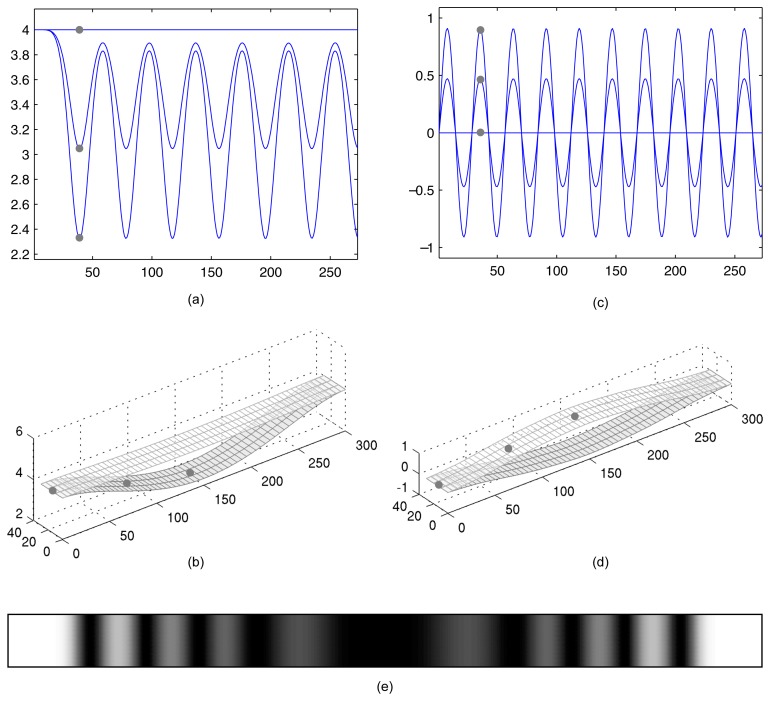
The formation of interference fringes for periodic and harmonic oscillation laws. Time graphs of three different points on the surface of the beam are shown in (**a**) for periodic and (**c**) harmonic oscillations. Maximum deformations of the FEM mesh are shown in (b) and (**d**). Time-averaged hologram of harmonic oscillation is given in (**e**).

As mentioned previously Figure 7a shows a well developed pattern of time averaged-holographic fringes. Nevertheless Figure 8a,b suggest that oscillation process of the beam is not harmonic (though it is periodic). Natural is the question if it is possible to reconstruct such harmonic oscillation of the beam that its time-averaged hologram would look almost identical to Figure 7a. In order to illustrate the complexity of the problem we do visualize maximum deflections of the beam when it does oscillate according to the data produced by computational experiment (i). Time graphs of three different points on the surface of the beam are shown in Figure 8a; maximum deformations of the FEM mesh—in Figure 8b.

Harmonic oscillations around the state of equilibrium (according to the first eigenmode of the beam) are illustrated in Figure 8c, d. Now we just need to preselect the maximum amplitude of harmonic oscillations in such a way that the generated pattern of the time-averaged holographic fringes would be as close as possible to the pattern in Figure 7a. It can be seen that the time-averaged hologram in Figure 8e is an almost identical copy of Figure 7a when the amplitude of harmonic oscillations of the central point of the beam is set to coordinates *x* = 150 and *y* = 10.

An important conclusion follows from the above observations. One should not use straightforward techniques for the identification of the process of the oscillation from the available image of time-averaged hologram. This inverse problem may become very complex if the motion law governing the dynamics of the analyzed structure is not known before the experiment.

### 5.5. The Formation of Interference Fringes for Different Time Intervals

The situation becomes even more complex when the oscillation is not periodic but is chaotic. As mentioned previously chaotic oscillations do not generate time-averaged holographic fringes. However, this result holds when exposure time tends to infinity. Finite exposure times result into interpretable patterns of fringes even for chaotic oscillations.

**Figure 9.**
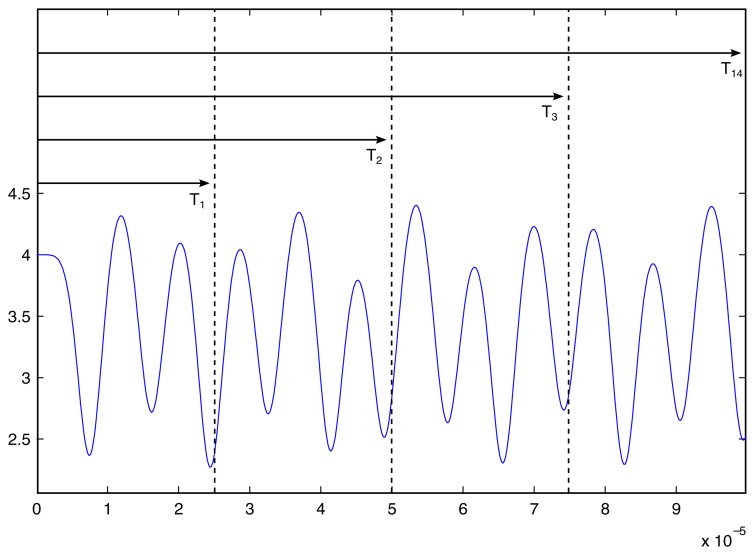
Computational experiment (iii) divided into time intervals (T1), (T2), (T3), (T4).

**Figure 10.**
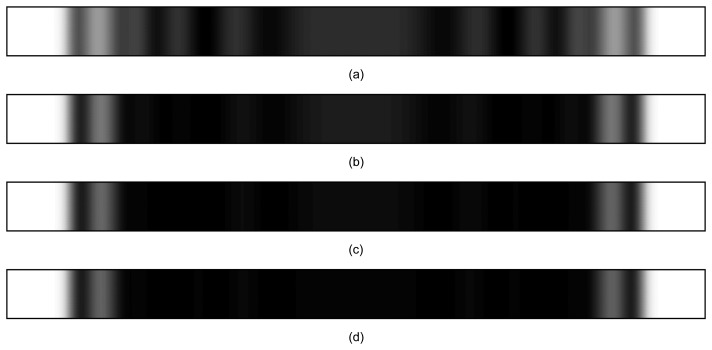
The formation of interference fringes for time intervals: (**a**) T1; (**b**) T2; (**c**) T3; (**d**) T4.

We use the same data produced by computational experiment (iii) but construct time-averaged holograms for different exposure times (Figure 9). Time-averaged holograms for exposure times *T*_1_, *T*_2_, *T*_3_ and *T*_4_ are illustrated in Figure 10. A gradual loss of the time-averaged fringes can be observed as exposure time increases. That suggests another important conclusion. A straightforward identification of the motion law from time-averaged holographic fringes is prone to severe errors if the motion law of the investigated structure is not known. A short exposure time over a chaotic motion (Figure 10a) suggests a harmonic oscillation of the fixed-fixed beam–though this is completely not true. Another important conclusion follows from Figure 10d. One should not blame his optical setup if the generated time-averaged hologram does not yield a pattern of fringes. The optical setup may be in perfect order—simply the structure may be oscillating according to a chaotic law.

## 6. Experiment

The methods of holographic interferometry allow to get much more information about the deformable surface comparing with other experimental methods. Because of this holographic interferometry methods are widely applied in mechanics, biology, non-destructive control, automotive industries and microsystem engineering and others areas.

Digital holography technology uses real-time, 3D, full field-of view surface measurements to eliminate point-by-point data gathering. Holograms are used to measure the surface of any component. Superb resolution allows for monitoring shape changes that are smaller than 20 nanometers for superb accuracy and resolution of the results

Measurements of vibration amplitudes of surface of MEMS, as functions of operating conditions of the MEMS were performed using the optoelectronic laser interferometric microscope. The methodology of investigation using optical micro-holographic system for recording resonance frequencies of vibrating mechatronic system is presented in paper [22].

**Figure 11.**
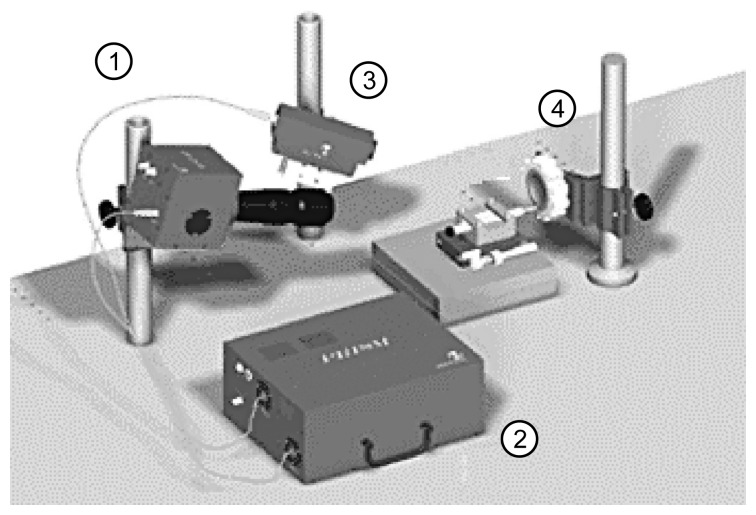
PRISMA system setup: (1) videohead; (2) control block; (3) illumination head of the object; (4) investigated object.

The general view of PRISM setup is presented in Figure 11. PRISM combines all the necessary equipment for deformation and vibration measurement of most materials in a small lightweight system. A standard system includes holography and computer systems integrated with proprietary state of the art software.

Success in deformation and vibration measurement requires a fast affordable solution. PRISM offers a high-speed holographic technique for production measurement of vibration and deformation without surface contact and minimal sample preparation. PRISM can access complex geometries (deep recesses or curves) difficult or impossible for other techniques and can be configured for specific needs in experimental investigation of mechatronics systems. The PRISM system videohead were modified in order to make registration microscale objects possible.

**Figure 12.**
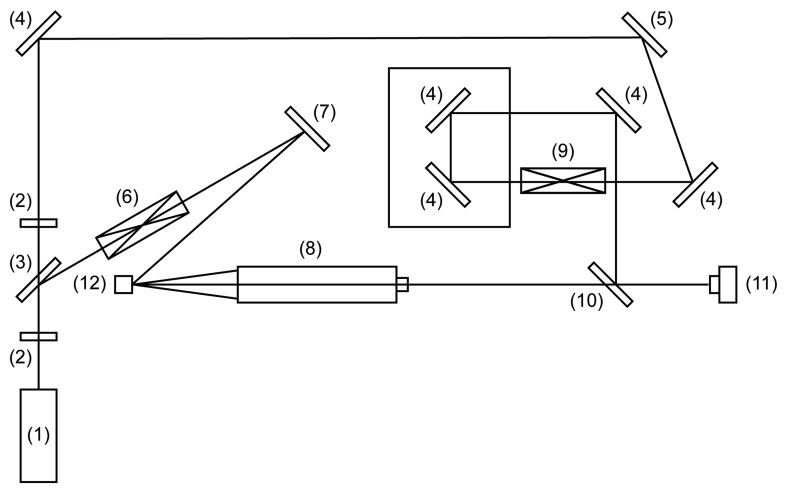
Schematic diagram of the microscopic holographic system: (1) He-Ne laser; (2) $\frac{\lambda }{2}$ plate (λ–length of the laser wavelength); (3) directional beam splitter (PBS); (4), (5), (7) mirrors; (6) spatial filter; (8) microscope; (9) telescope; (10) proximal beam splitter; (11) CCD camera; (12) analyzed object MEMS.

**Figure 13.**
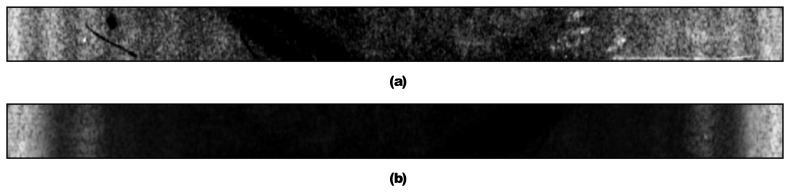
Holographic interferogram of vibrating MEMS, when fixed-fixed beam is performing: (**a**) periodic oscillations; (**b**) chaotic oscillations.

In Figure 12 optical micro holographic system for recording resonance frequencies of MEMS is illustrated. A coherent laser beam is passed through λ/2 plate (2). It then passes to a directional beam splitter where one part through spatial filter (6) and mirror (7) illuminates investigated MEMS (12) and mirror to MEMS device (12). The other through optical elements (2), (4), (5), (9), (10) are directed to CCD (11). Consequently, from the observation point, two intensity distributions are viewed: one of the light reflected from the object (12) and second arising from reflection off the bottom surface of proximal beam splitter (10). The reflected light from MEMS is transmitted back through the long distance microscope objective (8) and proximal beam splitter (10) to a digital camera CCD (11). Images are recorded by the camera of vibrating fixed-fixed beam which is performing periodic and chaotic oscillations. The results are given in Figure 13. These experiments were conducted according to Sections 3 and 4 of this article. It can be seen from Figure 13 that results correspond well with computational results in Section 5.3—decay of intensity is obvious in part (b) when fixed-fixed beam is performing chaotic oscillations.

The wave fronts are planar in the setup that used in this study, thus parallel interference fringes result when the proximal beam splitter is introduced into the path. Single frame images captured by the CCD and proceeded by computer give us a valuable insight in the motion of investigated MEMS.

## 7. Conclusions

Time averaged holography is a powerful optical experimental technique for the investigation of microsystem components. The inverse problem for the interpretation of time-averaged holographic fringes is a well defined and straightforward task, if only the oscillations of the investigated structures are harmonic. It is demonstrated that complex chaotic motions can be generated even in rather simple micro-electromechanical systems. Therefore, a straightforward interpretation of time-averaged holographic interferograms of micro-mechanical components can be not only be misguiding but the experimental holographic images may not reveal any interpretable fringes at all.

A relationship between the decay of intensity in a time-averaged holographic interferogram and the intensity of chaotic motion is explicitly derived. It is shown that chaotic oscillations do not generate time-averaged holographic fringes. Thus one needs to make sure that the response of the excited electro-mechanical system is not chaotic before questioning the functionality of the optical experimental setup. The derived analytical results yield a deeper insight into the dynamical properties of MEMS components.
